# A primal-dual algorithm framework for convex saddle-point optimization

**DOI:** 10.1186/s13660-017-1548-z

**Published:** 2017-10-25

**Authors:** Benxin Zhang, Zhibin Zhu

**Affiliations:** 1 School of Electronic Engineering and Automation, Guangxi Key Laboratory of Automatic Detecting Technology and Instruments, Guilin University of Electronic Technology, Jinji Road, Guilin, China; 2 School of Mathematics and Computing Science, Guangxi Colleges and Universities Key Laboratory of Data Analysis and Computation, Guilin University of Electronic Technology, Jinji Road, Guilin, 541004 China

**Keywords:** primal-dual method, proximal point algorithm, convex optimization, variational inequalities

## Abstract

In this study, we introduce a primal-dual prediction-correction algorithm framework for convex optimization problems with known saddle-point structure. Our unified frame adds the proximal term with a positive definite weighting matrix. Moreover, different proximal parameters in the frame can derive some existing well-known algorithms and yield a class of new primal-dual schemes. We prove the convergence of the proposed frame from the perspective of proximal point algorithm-like contraction methods and variational inequalities approach. The convergence rate $O(1/t)$ in the ergodic and nonergodic senses is also given, where *t* denotes the iteration number.

## Introduction

We consider the following model that arises from various signal and image processing applications:

1 $$ \min_{x} f_{1}(Bx)+f_{2}(x), $$

where *B* is a continuous linear operator, and $f_{1}$ and $f_{2}$ are proper convex lower-semicontinuous functions. We can easily write problem (1) in its primal-dual formulation through Fenchel duality [1]:

2 $$ \min_{x\in X}\max_{{v}\in V}L(x,{v}):=f_{2}(x)+ \langle Bx,{v}\rangle-f_{1}^{*}({v}), $$

where $X\in R^{N}$ and $V\in R^{M}$ are two finite-dimensional vector spaces, and $f_{1}^{*}$ is the convex conjugate function of $f_{1}$ defined as

$$f_{1}^{*}(v)=\sup_{{\omega}\in R^{M}}\langle{v},{\omega}\rangle -f_{1}({\omega}). $$

As analyzed in [2, 3], the saddle-point problem (2) can be regarded as the primal-dual formulation, and more and more scholars have proposed some primal-dual algorithms. Zhu and Chan [4] proposed the famous primal-dual hybrid gradient (PDHG) algorithm with adaptive stepsize. Though the algorithm is quite fast, the convergence is not proved. He, You, and Yuan [5] showed that PDHG with constant step sizes is indeed convergent if one of the functions of the saddle-point problem is strongly convex. Chambolle and Pock [2] gave a primal-dual algorithm with convergence rate $O(1/k)$ for the complete class of these problems. They further showed accelerations of the proposed algorithm to yield improved rates on problems with some degree of smoothness. In particular, they showed that the algorithm can achieve the $O(1/k^{2})$ convergence in problems where the primal or the dual objective is uniformly convex, and the method can show linear convergence, that is, $O(\varsigma^{k})$ (for some $\varsigma\in (0,1 )$), on smooth problems. Bonettini and Ruggiero [6] established the convergence of a general primal-dual method for nonsmooth convex optimization problems and showed that the convergence of the scheme can be considered as an *ϵ*-subgradient method on the primal formulation of the variational problem when the steplength parameters are a priori selected sequences. He and Yuan [7] did a novel study on these primal-dual algorithms from the perspective of contraction perspective. Their method simplified the existing convergence analysis. Cai, Han, and Xu [8] proposed a new correction strategy for some first-order primal-dual algorithms. Later, He, Desai, and Wang [9] introduced another new primal-dual prediction-correction algorithm for solving a saddle-point optimization problem, which serves as a bridge between the algorithms proposed in [8] and [7]. Recently, Zhang, Zhu, and Wang [10] proposed a simple primal-dual method for total-variation image restoration problems and showed that their iterative scheme has the $O(1/k)$ convergence rate in the ergodic sense. When we had finished this paper, we found the algorithm proposed in [11], where convergence analysis was similar to our proposed frame. However, the algorithm proposed in [11] is actually a particular case of our unified framework when the precondition matrix in our frame is fixed.

More specifically, the iterative schemes of existing primal-dual algorithms for the problem (2) can be unified as the following procedure:

3 $$ \textstyle\begin{cases} {v}_{k+1}=\arg\min_{{v}\in R^{M}} -L(x_{k},{v})+\frac{1}{2\gamma}\|{v}-{v}_{k}\|^{2}_{2}, \\ y_{k+1}={v}_{k+1}+\theta({v}_{k+1}-v_{k}), \\ x_{k+1}=\arg\min_{x\in R^{N}} L(x,y_{k+1})+\frac{1}{2\tau}\|x-x_{k}\|^{2}_{2}, \end{cases} $$

where $\gamma,\tau>0$ and $\theta\in R$. The combination parameter *θ* is set to zero in the original PDHG algorithm. When $\theta\in [0,1]$, the primal-dual algorithm proposed in [2] was recovered. He and Yuan [7] showed that the range of the combination parameter *θ* can be enlarged to $[-1,1]$. Komodakis and Pesquet [12] recently wrote a wonderful overview of recent primal-dual method for solving large-scale optimization problems. So, we refer the reader to [12] for more details.

In some imaging applications, for example, partially parallel magnetic resonance imaging [13], the primal subproblem in (3) may not be easy to solve. Because of this difficulty, it is advisable to use inner iterations to get approximate solutions of the subproblems. In the recent work, several completely decoupled schemes are proposed to avoid subproblem solving, such as primal-dual fixed point algorithm [14–16] and the Uzawa method [17]. Hence, motivated by the works [7, 10, 17], we reconsider the popular iterative scheme (3) and give a primal-dual algorithm framework such that it can be well adopted in different imaging applications.

The organization of this paper is as follows. In Section 2, we propose the primal-dual-based contraction algorithm framework in prediction-correction fashion. In Section 3, we present convergence analysis. The iteration complexity in the ergodic and nonergodic senses is established in Sections 4 and 5. In Section 6, connections with well-known methods, and some new schemes are discussed. Finally, a conclusion is given.

## Proposed frame

Problem (2) can be reformulated as the following monotone variational inequality (VI): Find $(x^{*},v^{*})\in X\times V$ such that

4 $$ \left ( \textstyle\begin{array}{@{}c@{}} v-v^{*} \\ x-x^{*} \end{array}\displaystyle \right )^{T} \left ( \textstyle\begin{array}{@{}c@{}} \partial f_{1}^{*}(v^{*})-Bx^{*} \\ \partial f_{2}(x^{*})+B^{T}v^{*} \end{array}\displaystyle \right )\geq0, \quad \forall(x,v)\in X \times V, $$

where *∂* denotes the subdifferential operator of a convex function. By denoting

$$u=\left ( \textstyle\begin{array}{@{}c@{}} v\\ x \end{array}\displaystyle \right ),\qquad F(u)=\left ( \textstyle\begin{array}{@{}c@{}} \partial f_{1}^{*}(v)-Bx \\ \partial f_{2}(x)+B^{T}v \end{array}\displaystyle \right ), \qquad \Omega=X\times V, $$

the VI (4) can be written as follows (denoted $\operatorname{VI}(\Omega,F)$):

$$\bigl(u-u^{*}\bigr)^{T}F\bigl(u^{*}\bigr)\geq0, \qquad \Omega=X\times V. $$

Note that the monotonicity of the variational inequality is guaranteed by the convexity of the function $\partial f_{1}^{*}$ and $\partial f_{2}$.

Recall that the primal-dual algorithm for (2) presented in [2] ($\theta=1$) is

5 $$ \textstyle\begin{cases} {v}_{k+1}=\arg\min_{{v}\in R^{M}} -L(x_{k},{v})+\frac{1}{2\gamma}\|{v}-{v}_{k}\|^{2}_{2}, \\ y_{k+1}=2{v}_{k+1}-v_{k}, \\ x_{k+1}=\arg\min_{x\in R^{N}} L(x,y_{k+1})+\frac{1}{2\tau}\|x-x_{k}\|^{2}_{2}. \end{cases} $$

We can easily verify that the iteration $(v_{k+1},x_{k+1})$ generated by (5) can be characterized as follows:

6 $$ \left ( \textstyle\begin{array}{@{}c@{}} v-v_{k+1}\\ x-x_{k+1} \end{array}\displaystyle \right )^{T} \left \{ \left ( \textstyle\begin{array}{@{}c@{}} \partial f_{1}^{*}(v_{k+1})-Bx_{k+1}\\ \partial f_{2}(x_{k+1})+B^{T}v_{k+1} \end{array}\displaystyle \right )+\left ( \textstyle\begin{array}{@{}c@{\quad}c@{}} \frac{1}{\gamma}I & B\\ B^{T} & \frac{1}{\tau}I \end{array}\displaystyle \right )\left ( \textstyle\begin{array}{@{}c@{}} v_{k+1}-v_{k}\\ x_{k+1}-x_{k} \end{array}\displaystyle \right ) \right \}\geq0. $$

The convergence of iteration (6) was proved in [2] with the condition on the stepsize $\gamma\tau\|B^{T}B\|<1$. Motivated by the idea in [7], the scheme (6) can be considered as a prediction step. So, in the following, we propose a primal-dual-based contraction method for problem (2). To present new methods in the prediction-correction fashion, we denote the iteration $\tilde{u}_{k}=(\tilde{v}_{k},\tilde{x}_{k})$ generated by the following primal-dual procedure (7), where the prediction step can be redescribed as

7 $$ \textstyle\begin{cases} \tilde{v}_{k}=\arg\min_{{v}\in R^{M}} -L(x_{k},{v})+\frac{1}{2\gamma}\|{v}-{v}_{k}\|^{2}_{2}, \\ \tilde{y}_{k}=2\tilde{v}_{k}-v_{k}, \\ \tilde{x}_{k}=\arg\min_{x\in R^{N}} L(x,\tilde{y}_{k})+\frac{1}{2\tau}\|x-x_{k}\|^{2}_{P}, \end{cases} $$

where *P* is a positive definite matrix to be selected properly in different applications. Then, the new iteration is yielded by correcting $\tilde{u}_{k}$ via

8 $$ u_{k+1}=u_{k}-\rho(u_{k}- \tilde{u}_{k}), $$

where $0<\rho<2$. Similarly to (6), the predictor scheme (7) can also be written in the VI form as follows:

9 $$ \left ( \textstyle\begin{array}{@{}c@{}} v-\tilde{v}_{k}\\ x-\tilde{x}_{k} \end{array}\displaystyle \right )^{T} \left \{ \left ( \textstyle\begin{array}{@{}c@{}} \partial f_{1}^{*}(\tilde{v}_{k})-B\tilde{x}_{k} \\ \partial f_{2}(\tilde{x}_{k})+B^{T}\tilde{v}_{k} \end{array}\displaystyle \right ) +\left ( \textstyle\begin{array}{@{}c@{\quad}c@{}} \frac{1}{\gamma}I&B\\ B^{T}& \frac{1}{\tau} P \end{array}\displaystyle \right )\left ( \textstyle\begin{array}{@{}c@{}} \tilde{v}_{k}-v_{k}\\ \tilde{x}_{k}-x_{k} \end{array}\displaystyle \right ) \right \}\geq0. $$

Setting

10 $$ Q= \left ( \textstyle\begin{array}{@{}c@{\quad}c@{}} \frac{1}{\gamma}I&B\\ B^{T}& \frac{1}{\tau} P \end{array}\displaystyle \right ) $$

and using the notation in (4), we have the following compact form of (9):

11 $$ (u-\tilde{u}_{k})^{T}\bigl\{ F( \tilde{u}_{k})+Q(\tilde{u}_{k}-u_{k})\bigr\} \geq0, \quad \forall u\in\Omega. $$

So, we can prove the convergence of the proposed algorithm in the form of proximal point algorithm [7, 18]. Next, we use this idea to prove that the scheme (7)-(8) converges.

## Convergence analysis

In this section, we show the convergence of the proposed frame. Convergence results easily follow from proximal point algorithm-like contraction methods [7] and VI approach [19].

### Lemma 1

*Let* *B* *be the given operator*, *let* $\gamma,\tau>0$, *and let* *Q* *be defined by* (10). *Then* *Q* *is positive definite if*

$$\gamma\tau \bigl\Vert B^{T}B \bigr\Vert < {p}, $$

*where* $p>0$ *is the minimal eigenvalue of* *P*.

### Proof

For any nonzero vectors *s* and *t*, we have

$$\begin{aligned}& \bigl(s^{T},t^{T}\bigr)\left ( \textstyle\begin{array}{@{}c@{\quad}c@{}} \frac{1}{\gamma}I & B\\ B^{T} & \frac{1}{\tau} P \end{array}\displaystyle \right )\left ( \textstyle\begin{array}{@{}c@{}} s\\ t \end{array}\displaystyle \right ) \\& \quad =\frac{ \Vert s \Vert ^{2}}{\gamma}+\frac{ \Vert t \Vert ^{2}_{P}}{\tau}+2s^{T}Bt \\& \quad \geq\frac{ \Vert s \Vert ^{2}}{\gamma}+\frac{p \Vert t \Vert ^{2}}{\tau}-2\sqrt{ \bigl\Vert B^{T}B \bigr\Vert } \Vert s \Vert \Vert t \Vert \\& \quad \geq2\biggl(\sqrt{\frac{p}{\gamma\tau}}-\sqrt{ \bigl\Vert B^{T}B \bigr\Vert }\biggr) \Vert s \Vert \Vert t \Vert , \end{aligned}$$

where we used the Cauchy-Schwarz inequality. The proof is completed. □

In the following, we give an important inequality for the output of the scheme (7)-(8).

### Lemma 2

*For iteration sequences* $\{u_{k}\}$ *and* $\{ \tilde{u}_{k}\}$, *we have*

12 $$ \bigl(u_{k}-u^{*}\bigr)^{T}Q(u_{k}- \tilde{u}_{k})\geq(u_{k}-\tilde{u}_{k})^{T}Q(u_{k}- \tilde{u}_{k}),\quad \forall u^{*}\in\Omega. $$

### Proof

Since (11) holds for any $u\in\Omega$, we set $u=u^{*}$, where $u^{*}$ is an arbitrary solution, and obtain

13 $$ \bigl(u^{*}-\tilde{u}_{k}\bigr)^{T}\bigl\{ F( \tilde{u}_{k})+Q(\tilde{u}_{k}-u_{k})\bigr\} \geq0. $$

Thus (13) leads to

14 $$ \bigl(u^{*}-\tilde{u}_{k}\bigr)^{T}Q( \tilde{u}_{k}-u_{k})\geq\bigl(\tilde{u}_{k}-u^{*} \bigr)^{T}F(\tilde{u}_{k}). $$

Note that the mapping $F(u)$ is monotone. We thus have

15 $$ \bigl(\tilde{u}_{k}-u^{*}\bigr)^{T}\bigl(F( \tilde{u}_{k})-F\bigl(u^{*}\bigr)\bigr)\geq0 $$

and also

$$\bigl(\tilde{u}_{k}-u^{*}\bigr)^{T}F(\tilde{u}_{k}) \geq\bigl(\tilde{u}_{k}-u^{*}\bigr)^{T}F\bigl(u^{*}\bigr)\geq0. $$

Replacing $u^{*}-\tilde{u}_{k}$ by $(u^{*}-u_{k})+(u_{k}-\tilde{u}_{k})$ in (14) and using (15), we get the assertion. □

### Lemma 3

*The sequence* $\{u_{k}\}$ *generated by the proposed scheme* (7)-(8) *satisfies*

16 $$ \bigl\Vert u_{k+1}-u^{*} \bigr\Vert ^{2}_{Q}\leq \bigl\Vert u_{k}-u^{*} \bigr\Vert ^{2}_{Q}-\rho(2-\rho) \Vert u_{k}- \tilde{u}_{k} \Vert ^{2}_{Q}, \quad \forall u^{*} \in\Omega. $$

### Proof

Using (8) and (12), by a simple manipulation we obtain

$$\begin{aligned} \bigl\Vert u_{k+1}-u^{*} \bigr\Vert ^{2}_{Q} =& \bigl\Vert u_{k}-u^{*}-\rho(u_{k}-\tilde{u}_{k}) \bigr\Vert ^{2} \\ =& \bigl\Vert u_{k}-u^{*} \bigr\Vert ^{2}_{Q}-2 \rho\bigl(u_{k}-u^{*}\bigr)^{T}Q(u_{k}- \tilde{u}_{k})+\rho^{2} \Vert u_{k}- \tilde{u}_{k} \Vert ^{2}_{Q} \\ \leq& \bigl\Vert u_{k}-u^{*} \bigr\Vert ^{2}_{Q}-2 \rho \Vert u_{k}-\tilde{u}_{k} \Vert ^{2}_{Q}+ \rho^{2} \Vert u_{k}-\tilde {u}_{k} \Vert ^{2}_{Q} \\ =& \bigl\Vert u_{k}-u^{*} \bigr\Vert ^{2}_{Q}- \rho(2-\rho) \Vert u_{k}-\tilde{u}_{k} \Vert ^{2}_{Q}. \end{aligned}$$

The assertion is proved. □

The following theorem states that the proposed iterative scheme converges to an optimal primal-dual solution.

### Theorem 1

*If* *Q* *in* (10) *is positive definite*, *then any sequence generated by the scheme* (7)-(8) *converges to a solution of the minimax problem* (2).

### Proof

From (16) we know that the norm $\|u_{k}-u^{*}\|_{Q}$ is nonincreasing. We also can get that $u_{k}$ is bounded and $\|u_{k}-\tilde{u}_{k}\|_{Q}\rightarrow0$. Inequality (16) implies that the sequence $\{u_{k}\}$ has at least one cluster point. We denote it by $u^{\infty}$. Let $\{u_{k_{j}}\}$ be a subsequence converging to $u^{\infty}$. Thus we have

17 $$ \lim_{j\rightarrow\infty}\|u_{k_{j}}- \tilde{u}_{k_{j}}\|=0. $$

Due to the facts (11) and (17), we have

$$\lim_{j\rightarrow\infty}\bigl(u-u^{k_{j}}\bigr)^{T}F(u_{k_{j}}) \geq0,\quad \forall u\in\Omega. $$

Because $\{u_{k_{j}}\}$ converges to $u^{\infty}$, this inequality becomes

$$\bigl(u-u^{\infty}\bigr)^{T}F\bigl(u^{\infty}\bigr)\geq0, \quad \forall u\in\Omega. $$

Thus, the cluster point $u^{\infty}$ satisfies the optimality condition of (2). Note that inequality (16) is true for all solution points of $\operatorname{VI}(\Omega,F)$. Hence we have

$$\bigl\Vert u_{k+1}-u^{\infty} \bigr\Vert \leq \bigl\Vert u_{k}-u^{\infty} \bigr\Vert , $$

and thus the sequence ${u_{k}}$ converges to $u^{\infty}$. This proof is completed. □

## Convergence rate in an ergodic sense

In the following, using proximal point algorithm-like contraction methods for convex optimization [19], the convergence rate in the ergodic and nonergodic senses is given. First, we prove a lemma, which is the base for the proofs of the convergence rate in the ergodic sense.

### Lemma 4

*The sequence* $\{u_{k}\}$ *is generated by the proposed scheme* (7)-(8). *Then we have*

18 $$ (u-\tilde{u}_{k})^{T}F({u})+\frac{1}{2\rho} \Vert u-u_{k} \Vert _{Q}^{2}\geq \frac{1}{2\rho } \Vert u-u_{k+1} \Vert _{Q}^{2}, \quad \forall u\in\Omega. $$

### Proof

Using (8), the right-hand side of (11) can be written as

19 $$ \rho(u-\tilde{u}_{k})^{T}F( \tilde{u}_{k})\geq(u-\tilde {u}_{k})^{T}Q(u_{k}-u_{k+1}), \quad \forall u\in\Omega. $$

For the right-hand side of (19), taking

$$a=u,\qquad b=\tilde{u}_{k},\qquad c=u_{k}, \qquad d=u_{k+1}, $$

and applying the identity

$$(a-b)^{T}Q(c-d)=\frac{1}{2}\bigl\{ \|a-d\|^{2}_{Q}- \|a-c\|^{2}_{Q}\bigr\} +\frac{1}{2}\bigl\{ \|c-b \|^{2}_{Q}-\| d-b\|^{2}_{Q}\bigr\} , $$

we obtain

20 $$ \begin{aligned}[b] (u-\tilde{u}_{k})^{T}Q(u_{k}-u_{k+1})={}& \frac{1}{2}\bigl\{ \|u-u_{k+1}\|^{2}_{Q}- \|u-u_{k}\| ^{2}_{Q}\bigr\} \\ &{}+\frac{1}{2}\bigl\{ \|u_{k}-\tilde{u}_{k}\|^{2}_{Q}- \|u_{k+1}-\tilde{u}_{k}\|^{2}_{Q}\bigr\} . \end{aligned} $$

For the last term of the right-hand side of (20), we have

21 $$\begin{aligned}& \Vert u_{k}-\tilde{u}_{k} \Vert ^{2}_{Q}- \Vert u_{k+1}-\tilde{u}_{k} \Vert ^{2}_{Q} \\& \quad = \Vert u_{k}-\tilde{u}_{k} \Vert ^{2}_{Q}- \bigl\Vert u_{k}-\tilde{u}_{k}-(u_{k}-{u}_{k+1}) \bigr\Vert ^{2}_{Q} \\& \quad = \Vert u_{k}-\tilde{u}_{k} \Vert ^{2}_{Q}- \bigl\Vert (1-\rho) (u_{k}- \tilde{u}_{k}) \bigr\Vert ^{2}_{Q} \\& \quad = \rho(2-\rho) \Vert u_{k}-\tilde{u}_{k} \Vert ^{2}_{Q}. \end{aligned}$$

Substituting (20) and (21) into (19), we get

22 $$ \rho(u-\tilde{u}_{k})^{T}F( \tilde{u}_{k})\geq\frac{1}{2}\bigl(\|u-u_{k+1} \|_{Q}^{2}-\| u-u_{k}\|_{Q}^{2} \bigr)+\frac{\rho(2-\rho)}{2} \|u_{k}-\tilde{u}_{k} \|_{Q}^{2}. $$

Using the property of the mapping *F*, we have

$$(u-\tilde{u}_{k})^{T}F({u})\geq(u-\tilde{u}_{k})^{T}F( \tilde{u}_{k}). $$

Substituting it into (22), the lemma is proved. □

### Theorem 2

*Let* $\{u_{k}\}$ *be the sequence generated by the scheme* (7)-(8), *and let* $\tilde{u}_{t}$ *be defined by*

23 $$ \tilde{u}_{t}=\frac{1}{t+1}\sum _{k=0}^{t}\tilde{u}_{k}. $$

*Then*, *for any integer* $t>0$, *we have that* $\tilde{u}_{t}\in\Omega$ *and*

24 $$ (\tilde{u}_{t}-u)^{T}F(u)\leq \frac{1}{2\rho(t+1)}\|u-u_{0}\|_{Q}^{2}, \quad \forall u\in\Omega. $$

### Proof

By the convexity of Ω it is clear that $\tilde{u}_{t}\in\Omega$. Summing (18) over $k=0, 1,\ldots, t$, we have

$$\Biggl((t+1)u-\sum_{k=0}^{t} \tilde{u}_{k} \Biggr)^{T}F(u)+\frac{1}{2\rho } \|u-u_{0}\|_{Q}^{2}\geq0,\quad \forall u\in\Omega. $$

By the definition of $\tilde{u}_{t}$, the assertion of the theorem directly follows. □

## Convergence rate in a nonergodic sense

In this section, we show that a worst-case $O(1/t)$ convergence rate in a nonergodic sense can also be established for the proposed algorithm frame. We first prove the following lemma.

### Lemma 5

*Let the sequence* $\{u_{k}\}$ *be generated by the proposed scheme* (7)-(8). *Then we have*

25 $$ (u_{k}-\tilde{u}_{k})^{T}Q\bigl\{ u_{k}-\tilde{u}_{k}-(u_{k+1}-\tilde{u}_{k+1}) \bigr\} \geq \frac{1}{2\rho} \bigl\Vert u_{k}-\tilde{u}_{k}-(u_{k+1}- \tilde{u}_{k+1}) \bigr\Vert _{Q^{T}+Q}^{2}. $$

### Proof

Setting $u=\tilde{u}_{k+1}$ in (11), we get

26 $$ (\tilde{u}_{k+1}-\tilde{u}_{k})^{T}F( \tilde{u}_{k})\geq(\tilde {u}_{k+1}-\tilde{u}_{k})^{T}Q(u_{k}- \tilde{u}_{k}). $$

Note that (11) is also true for $k :=k+1$, and we have

$$(u-\tilde{u}_{k+1})^{T}F(\tilde{u}_{k+1})\geq({u}- \tilde {u}_{k+1})^{T}Q(u_{k+1}-\tilde{u}_{k+1}), \quad \forall u\in\Omega. $$

Setting $u=\tilde{u}_{k}$ in this inequality, we obtain

27 $$ (\tilde{u}_{k}-\tilde{u}_{k+1})^{T}F( \tilde{u}_{k+1})\geq(\tilde {u}_{k}-\tilde{u}_{k+1})^{T}Q(u_{k+1}- \tilde{u}_{k+1}). $$

Adding (26) and (27) and using the monotonicity of *F*, we obtain

28 $$ (\tilde{u}_{k}-\tilde{u}_{k+1})^{T}Q \bigl\{ ({u}_{k}-\tilde{u}_{k})-(u_{k+1}-\tilde {u}_{k+1})\bigr\} \geq0. $$

Adding the term

$$\bigl\{ ({u}_{k}-\tilde{u}_{k})-(u_{k+1}- \tilde{u}_{k+1})\bigr\} ^{T}Q\bigl\{ ({u}_{k}-\tilde {u}_{k})-(u_{k+1}-\tilde{u}_{k+1})\bigr\} $$

to both sides of (28), we have

$$({u}_{k}-{u}_{k+1})^{T}Q\bigl\{ ({u}_{k}- \tilde{u}_{k})-(u_{k+1}-\tilde{u}_{k+1})\bigr\} \geq \frac{1}{2} \bigl\Vert u_{k}-\tilde{u}_{k}-(u_{k+1}- \tilde{u}_{k+1}) \bigr\Vert _{Q^{T}+Q}^{2}. $$

Substituting $u_{k}-u_{k+1}=\rho(u_{k}-\tilde{u}_{k})$ into the left-hand side of the inequality, we obtain the lemma. □

Next, we are ready to prove the key inequality of this section.

### Lemma 6

*Let the sequence* $\{u_{k}\}$ *be generated by the proposed scheme* (7)-(8). *Then we have*

29 $$ \Vert u_{k+1}-\tilde{u}_{k+1} \Vert _{Q}^{2}\leq \Vert u_{k}-\tilde{u}_{k} \Vert ^{2}_{Q}. $$

### Proof

Taking $a=u_{k}-\tilde{u}_{k}$, $b=u_{k+1}-\tilde {u}_{k+1}$ in the identity

$$\|a\|^{2}_{Q}-\|b\|^{2}_{Q}=2a^{T}Q(a-b)- \|a-b\|^{2}_{Q}, $$

we have

$$\begin{aligned}& \Vert u_{k}-\tilde{u}_{k} \Vert ^{2}_{Q}- \Vert u_{k+1}-\tilde{u}_{k+1} \Vert ^{2}_{Q} \\& \quad = 2(u_{k}-\tilde{u}_{k})^{T}Q \bigl((u_{k}-\tilde{u}_{k})-(u_{k+1}- \tilde{u}_{k+1})\bigr) - \bigl\Vert (u_{k}- \tilde{u}_{k})-(u_{k+1}-\tilde{u}_{k+1}) \bigr\Vert ^{2}_{Q}. \end{aligned}$$

Since inequality (25) holds, we obtain

$$\begin{aligned} \begin{aligned} &\Vert u_{k}-\tilde{u}_{k} \Vert ^{2}_{Q}- \Vert u_{k+1}-\tilde{u}_{k+1} \Vert ^{2}_{Q} \\ &\quad \geq \frac{2-\rho}{\rho} \bigl\Vert (u_{k}- \tilde{u}_{k})-(u_{k+1}-\tilde{u}_{k+1}) \bigr\Vert ^{2}_{Q}\geq0. \end{aligned} \end{aligned}$$

The assertion directly follows from this inequality. □

Now, we establish a worst-case $O(1/t)$ convergence rate in a nonergodic sense.

### Theorem 3

*Let* $\{u_{k}\}$ *be the sequence generated by the scheme* (7)-(8). *Then*, *for any integer* $t>0$, *we have*

30 $$ \Vert u_{t}-\tilde{u}_{t} \Vert _{Q}^{2}\leq\frac{1}{\rho(2-\rho)(t+1)} \bigl\Vert u_{0}-u^{*} \bigr\Vert _{Q}^{2},\quad \forall u \in\Omega. $$

### Proof

It follows from (16) that

31 $$ \sum_{k=0}^{\infty}\rho(2-\rho) \Vert u_{k}-\tilde{u}_{k} \Vert ^{2}_{Q} \leq \bigl\Vert u_{0}-u^{*} \bigr\Vert _{Q}^{2}, \quad \forall u^{*}\in\Omega. $$

By Lemma 6 the sequence $\{\|u_{k}-\tilde{u}_{k}\|_{Q}^{2}\}$ is nonincreasing. So, we obtain

32 $$ (t+1) \Vert u_{k}-\tilde{u}_{k} \Vert ^{2}_{Q}\leq\sum_{k=0}^{t} \Vert u_{k}-\tilde{u}_{k} \Vert ^{2}_{Q}. $$

Assertion (30) immediately follows from (31) and (32). □

## Connections with existing methods

In this section, we focus on a specific version of problem (1),

33 $$ \min_{x} f_{1}(Bx)+ \frac{1}{2}\|Ax-b\|^{2}, $$

which arises in imaging processing, where $f_{2}(x)=\frac{1}{2}\|Ax-b\|^{2}$ is quadratic. For discrete total-variation regularization, *B* is the gradient operator, and *A* is a possibly large and ill-conditioned matrix representing a linear transform. If *A* is the identity matrix, then problem (1) is the well-known Rudin-Osher-Fatemi denoising model [20]. Because total-variation regularization can preserve sharp discontinuities in an image for removing noise, the above problem has received a lot of attention by most scholars in image processing, including computerized tomography [14] and parallel magnetic resonance imaging [13].

In the following, we establish connections of the proposed frame to the well-known methods for solving (33). There are other types methods designed to solve problem (33). Among them, the split Bregman method proposed by Goldstein and Osher [21] is very popular for imaging applications. This method has been proved to be equivalent to the alternating direction of multiplier method. In [17], based on proximal forward-backward splitting and Bregman iteration, a split inexact Uzawa (SIU) method is proposed to maximally decouple the iterations, so that each iteration is explicit in this algorithm. Also, the authors gave an algorithm based on Bregman operator splitting (BOS) when *A* is not diagonalizable. Recently, Tian and Yuan [11] proposed a linearized primal-dual method for linear inverse problems with total-variation regularization and showed that this variant yields significant computational benefits. Next, we show that different *P* in (7) can induce the following well-known methods: the linearized primal-dual method, SIU, BOS, and split Bregman methods and some new primal-dual algorithms with the correction step (8).

### Linearized primal-dual method

The linearized primal-dual method in [11] can be directly induced by setting $P=I-\tau A^{T}A$ and

34 $$ Q= \left ( \textstyle\begin{array}{@{}c@{\quad}c@{}} \frac{1}{\gamma}I&B\\ B^{T}& \frac{1}{\tau}I-A^{T}A \end{array}\displaystyle \right ). $$

We can also easily show that the positive definiteness of the matrices *P* and *Q* in (34) is guaranteed if $\gamma>0$, $0<\tau<1/\|A^{T}A\| $, $0<\tau<1/\|A^{T}A+\gamma B^{T}B\|$. In this situation, the scheme (7) can be written as follows:

35 $$ \textstyle\begin{cases} \tilde{v}_{k}=(I+\gamma\partial f_{1}^{*})^{-1}(v_{k}+\gamma Bx_{k}), \\ \tilde{x}_{k} =x_{k}-\tau\nabla f_{2}(x_{k})-\tau B^{T} (2\tilde{v}_{k}-v_{k}), \end{cases} $$

and the scheme (8) can be expressed as

36 $$ \textstyle\begin{cases} x_{k+1}=x_{k}-\rho(x_{k}-\tilde{x}_{k}), \\ v_{k+1}=v_{k}-\rho(v_{k}-\tilde{v}_{k}). \end{cases} $$

The idea is also similar to that of [22, 23], which uses the symmetric positive semi-definite matrix instead of the identity matrix in the proximal term. But their methods [22, 23] do not have overrelaxation or correction step. In [24, 25], the authors developed first-order splitting algorithm for solving jointly the primal and dual formulations of large-scale convex minimization problems involving the sum of a smooth function with Lipschitzian gradient, a nonsmooth proximable function, and linear composite functions. Actually, the linearized primal-dual method (35) and (36) is a particular case where a nonsmooth proximable function is missing in [24, 25].

When $\rho=1$, we can see that there is no correction step, that is, $(x_{k+1},v_{k+1})=(\tilde{x}_{k}, \tilde{v}_{k})$. In the following subsection, we focus on the scheme (7)-(8) with different *P* and *Q* when $\rho=1$, that is,

37 $$ \textstyle\begin{cases} {v}_{k+1}=\arg\min_{{v}\in R^{M}} -L(x_{k},{v})+\frac{1}{2\gamma}\|{v}-{v}_{k}\|^{2}_{2}, \\ y_{k+1}=2{v}_{k+1}-v_{k}, \\ x_{k+1}=\arg\min_{x\in R^{N}} L(x,y_{k+1})+\frac{1}{2\tau}\|x-x_{k}\|^{2}_{P}. \end{cases} $$

If $P=I$, the the CP method is a particular case of (37) as discussed in [7]. We also find that different *P* in (37) can induce some existing famous algorithms.

### Split inexact Uzawa method

For $f_{2}(x)=\frac{1}{2}\|Ax-b\|^{2}$, the explicit SIU algorithm can be described as follows:

38 $$ \textstyle\begin{cases} x_{k+1}=x_{k}-\tau A^{T}(Ax_{k}-b)-\tau\gamma B^{T}(Bx_{k}-d_{k}+\frac{v_{k}}{\gamma}),\\ d_{k+1}=\operatorname{prox}_{\frac{1}{\gamma}f_{1}}(Bx_{k+1}+\frac{v_{k}}{\gamma}),\\ {v}_{k+1}=v_{k}+\gamma(Bx_{k+1}-d_{k+1}), \end{cases} $$

where $\gamma>0$, $0<\tau<1/\|A^{T}A+\gamma B^{T}B\|$, and

$$\operatorname{prox}_{\frac{1}{\gamma}} f(v)=\arg\min f_{1}(v)+ \frac{1}{2\gamma}\|v-z\|^{2}_{2},\quad z\in V. $$

Let $P=I- \tau A^{T}A$ in (37). Then

$$Q= \left ( \textstyle\begin{array}{@{}c@{\quad}c@{}} \frac{1}{\gamma}I&B\\ B^{T}& \frac{1}{\tau}I-A^{T}A \end{array}\displaystyle \right ), $$

where $\gamma>0$, $0<\tau<1/\|A^{T}A\|$, $0<\tau<1/\|A^{T}A+\gamma B^{T}B\|$. So, the scheme (37) can be expressed as

39 $$ \textstyle\begin{cases} {v}_{k+1}=(I+\gamma\partial f_{1}^{*})^{-1}(v_{k}+\gamma Bx_{k}), \\ y_{k+1}=2{v}_{k+1}-v_{k}, \\ x_{k+1}=x_{k}-\tau\nabla f_{2}(x_{k})-\tau B^{T} y_{k+1}. \end{cases} $$

Using the relation $\operatorname{prox}_{\gamma f_{1}^{*}}=(I+\gamma\partial f_{1}^{*})^{-1}$ and changing the order of these equations, the scheme (39) is equivalent to

40 $$ \textstyle\begin{cases} x_{k+1}=x_{k}-\tau A^{T}(Ax_{k}-b)-\tau B^{T}(2{v}_{k}-v_{k-1}), \\ {v}_{k+1}=\operatorname{prox}_{\gamma f_{1}^{*}}(v_{k}+\gamma Bx_{k+1}). \end{cases} $$

By the Moreau decomposition (see equation (2.21) in [26]), for all $v\in R^{M}$ and $\lambda>0$, we have

$$v=\operatorname{prox}_{\lambda f}(v)+\lambda \operatorname{prox}_{\frac{1}{\lambda}f^{*}}(v/ \lambda). $$

Then

41 $$ \textstyle\begin{cases} x_{k+1}=x_{k}-\tau A^{T}(Ax_{k}-b)-\tau B^{T}(2{v}_{k}-v_{k-1}), \\ {v}_{k+1}=v_{k}+\gamma Bx_{k+1}-\gamma \operatorname{prox}_{\frac{1}{\gamma} f_{1}}(Bx_{k+1} +\frac{v_{k}}{\gamma}). \end{cases} $$

By introducing the variable $d_{k+1}=\operatorname{prox}_{\frac{1}{\gamma }f_{1}}(Bx_{k+1}+\frac{v_{k}}{\gamma})$, the scheme (41) can be further expressed as

42 $$ \textstyle\begin{cases} x_{k+1}=x_{k}-\tau A^{T}(Ax_{k}-b)-\tau B^{T}(2{v}_{k}-v_{k-1}), \\ d_{k+1}=\operatorname{prox}_{\frac{1}{\gamma}f_{1}}(Bx_{k+1}+\frac{v_{k}}{\gamma}), \\ {v}_{k+1}=v_{k}+\gamma(Bx_{k+1}-d_{k+1}). \end{cases} $$

Noting that ${v}_{k}=v_{k-1}+\gamma(Bx_{k}-d_{k})$, we have

43 $$ 2{v}_{k}-v_{k-1}=v_{k}+ \gamma(Bx_{k}-d_{k}). $$

Substituting (43) into the first equation of (42), the scheme (42) is equivalent to

44 $$ \textstyle\begin{cases} x_{k+1}=x_{k}-\tau A^{T}(Ax_{k}-b)-\tau\gamma B^{T}(Bx_{k}-d_{k}+\frac{v_{k}}{\gamma}), \\ d_{k+1}=\operatorname{prox}_{\frac{1}{\gamma}f_{1}}(Bx_{k+1}+\frac{v_{k}}{\gamma}), \\ {v}_{k+1}=v_{k}+\gamma(Bx_{k+1}-d_{k+1}). \end{cases} $$

We can see that the method (44) is equivalent to the SIU. Obviously, the explicit SIU method is a particular case of the proposed frame with $P=I-\tau A^{T}A$ and $\rho=1$. If $\rho\neq1$, then a linearized primal-dual method is presented in Section 6.1. So, the algorithm in [11] can be considered as a relaxed SIU method.

### Bregman operator splitting

The BOS algorithm for solving problem (33) was recently introduced in [17] based on the primal dual formulation of the model. It can be described as

45 $$ \textstyle\begin{cases} x_{k+1}=(\frac{1}{\tau}I+\gamma B^{T}B)^{-1}(\frac{1}{\tau }x_{k}- A^{T}(Ax_{k}-b)+\gamma B^{T}(d_{k}-\frac{v_{k}}{\gamma})), \\ d_{k+1}=\operatorname{prox}_{\frac{1}{\gamma}f_{1}}(Bx_{k+1}+\frac{v_{k}}{\gamma}), \\ {v}_{k+1}=v_{k}+\gamma(Bx_{k+1}-d_{k+1}), \end{cases} $$

where $\gamma>0$, $0<\tau\|A^{T}A\|<1$.

Similarly, let $P=I-\tau A^{T}A+\tau\gamma B^{T}B$ in (37). Then

$$Q= \left ( \textstyle\begin{array}{@{}c@{\quad}c@{}} \frac{1}{\gamma}I&B\\ B^{T}& \frac{1}{\tau}I- A^{T}A+\gamma B^{T}B \end{array}\displaystyle \right ), $$

where $\gamma>0$, $0<\tau<1/\|A^{T}A\|$, $0<\tau<1/\|A^{T}A-\gamma B^{T}B\|$. The scheme (37) can be expressed as

46 $$ \textstyle\begin{cases} x_{k+1} =(\frac{1}{\tau}I+\gamma B^{T}B)^{-1}((\frac{1}{\tau}I-A^{T}A+\gamma B^{T}B)x_{k}-B^{T}(2v_{k}-v_{k-1})+A^{T}b), \\ d_{k+1}=\operatorname{prox}_{\frac{1}{\gamma}f_{1}}(Bx_{k+1}+\frac{v_{k}}{\gamma}), \\ {v}_{k+1}=v_{k}+\gamma(Bx_{k+1}-d_{k+1}). \end{cases} $$

Using relation (43), we arrive at

47 $$ \textstyle\begin{cases} x_{k+1}=(\frac{1}{\tau}I+\gamma B^{T}B)^{-1}(\frac{1}{\tau }x_{k}- A^{T}(Ax_{k}-b)+\gamma B^{T}(d_{k}-\frac{v_{k}}{\gamma})), \\ d_{k+1}=\operatorname{prox}_{\frac{1}{\gamma}f_{1}}(Bx_{k+1}+\frac{v_{k}}{\gamma}), \\ {v}_{k+1}=v_{k}+\gamma(Bx_{k+1}-d_{k+1}). \end{cases} $$

Now, we can see that the scheme (47) is the method (45). Clearly, the iterative scheme (45) is a particular case of the frame with $P=I-\tau A^{T}A+\tau\gamma B^{T}B$ and $\rho=1$. Also, when $\rho\neq1$, we can get a new primal-dual method for solving (33) as follows:

48 $$ \textstyle\begin{cases} \tilde{v}_{k}=(I+\gamma\partial f_{1}^{*})^{-1}(v_{k}+\gamma Bx_{k}), \\ \tilde{x}_{k} =(\frac{1}{\tau}+\gamma B^{T}B)^{-1}((\frac{1}{\tau}-A^{T}A+\gamma B^{T}B)x_{k}-B^{T}(2\tilde{v}_{k}-v_{k})+A^{T}b), \\ x_{k+1}=x_{k}-\rho(x_{k}-\tilde{x}_{k}), \\ v_{k+1}=v_{k}-\rho(v_{k}-\tilde{v}_{k}). \end{cases} $$

In fact, the scheme (48) can be considered as a relaxed BOS algorithm. If $f_{1}(\cdot)=\|\cdot\|_{1}$, then we can deduce that $(I+\partial f_{1}^{*})^{-1}(v)=\operatorname{proj}(v)$, where proj is the projection operator. If the image satisfies periodic boundary conditions and if we use total-variation regularization, then the matrix $B^{T}B$ is block circulant; hence, it can be diagonalized by the Fourier transform matrix as noted in [27]. So, the new algorithm (48) can be computed efficiently and does not need the inner iteration to solve the subproblem.

### Split Bregman

In this subsection, we identify the split Bregman algorithm as a particular case of the proposed algorithm. Firstly, we reformulate model (33) as an equivalent constrained minimization problem

$$\min_{x,d}\bigl\{ f_{1}(d)+f_{2}(x): Bx-d=0\bigr\} . $$

The split Bregman algorithm for solving this constrained problem is as follows:

$$\textstyle\begin{cases} x_{k+1}=\arg\min_{x} f_{2}(x)+\langle v_{k},Bx-d_{k}\rangle +\frac{\gamma}{2}\|Bx-d_{k}\|^{2}, \\ d_{k+1}=\arg\min_{d} f_{1}(d)+\langle v_{k},Bx_{k+1}-d\rangle+\frac {\gamma}{2}\|Bx_{k+1}-d\|^{2}, \\ {v}_{k+1}=v_{k}+\gamma(Bx_{k+1}-d_{k+1}). \end{cases} $$

When $f_{2}(x)=\frac{1}{2}\|Ax-b\|^{2}$, it can be also described as

49 $$ \textstyle\begin{cases} x_{k+1}=(A^{T}A+\gamma B^{T}B)^{-1}(A^{T}b+ B^{T}(\gamma d_{k}-v_{k})), \\ d_{k+1}=\operatorname{prox}_{\frac{1}{\gamma}f_{1}}(Bx_{k+1}+\frac{v_{k}}{\gamma}), \\ {v}_{k+1}=v_{k}+\gamma(Bx_{k+1}-d_{k+1}), \end{cases} $$

where $\gamma>0$. The difficulty of implementing the scheme (49) is mainly due to that the inverse of the matrix is not easy to obtain. Next, we show that our method can induce the scheme (49).

Let

$$Q= \left ( \textstyle\begin{array}{@{}c@{\quad}c@{}} \frac{1}{\gamma}I&B\\ B^{T}& \gamma B^{T}B \end{array}\displaystyle \right ), $$

where $\tau=1$, $\gamma>0$. We see that the matrix is not positive. But the scheme (37) with this *Q* can induce the famous split Bregman algorithm. In this situation, the scheme (37) can be expressed as

50 $$ \textstyle\begin{cases} x_{k+1}=(A^{T}A+\gamma B^{T}B)^{-1}(A^{T}b- B^{T}(2v_{k}-v_{k-1})+\gamma B^{T}Bx_{k}), \\ d_{k+1}=\operatorname{prox}_{\frac{1}{\gamma}f_{1}}(Bx_{k+1}+\frac{v_{k}}{\gamma}), \\ {v}_{k+1}=v_{k}+\gamma(Bx_{k+1}-d_{k+1}). \end{cases} $$

Using the relation

$$2{v}_{k}-v_{k-1}=v_{k}+\gamma(Bx_{k}-d_{k}), $$

the scheme (50) can be further expressed as

51 $$ \textstyle\begin{cases} x_{k+1}=(A^{T}A+\gamma B^{T}B)^{-1}(A^{T}b+ B^{T}(\gamma d_{k}-v_{k})), \\ d_{k+1}=\operatorname{prox}_{\frac{1}{\gamma}f_{1}}(Bx_{k+1}+\frac{v_{k}}{\gamma}), \\ {v}_{k+1}=v_{k}+\gamma(Bx_{k+1}-d_{k+1}). \end{cases} $$

The method (51) is the split Bregman scheme (49). So, the split Bregman algorithm can be identified as a particular case of our proposed algorithm framework with $P=\gamma B^{T}B$ and $\rho=1$. If $\rho\neq1$, then, for $P=\gamma B^{T}B$ and $\tau=1$, a new primal-dual scheme can be described as follows:

52 $$ \textstyle\begin{cases} \tilde{v}_{k}=(I+\gamma\partial f_{1}^{*})^{-1}(v_{k}+\gamma Bx_{k}), \\ \tilde{x}_{k}=(A^{T}A+\gamma B^{T}B)^{-1}(\gamma B^{T}Bx_{k}-B^{T}(2\tilde {v}_{k}-v_{k-1})+A^{T}b), \\ x_{k+1}=x_{k}-\rho(x_{k}-\tilde{x}_{k}), \\ v_{k+1}=v_{k}-\rho(v_{k}-\tilde{v}_{k}). \end{cases} $$

Because the matrix *Q* is not positive definite, the split Bregman method may be not convergent. This case was also discussed in [16] with the same result. Based on Lemma 1, it suffices to replace the matrix *Q* by its perturbation in positive definite style. Similarly to [16], we modified the matrix *Q* as

53 $$ Q= \left ( \textstyle\begin{array}{@{}c@{\quad}c@{}} \frac{1}{\gamma}I&B\\ B^{T}& \gamma\theta B^{T}B+\alpha(1-\theta)I \end{array}\displaystyle \right ), $$

where *α* is a positive number, and *θ* is a number between 0 and 1. Using a similar derivation as before, the modified split Bregman algorithm is

54 $$ \textstyle\begin{cases} x_{k+1}=(A^{T}A+\gamma\theta B^{T}B+\alpha(1-\theta )I)^{-1}((1-\theta)(\alpha-\gamma B^{T}B)x_{k} \\ \hphantom{x_{k+1}={}}{}+A^{T}b+ B^{T}(\gamma d_{k}-v_{k})), \\ d_{k+1}=\operatorname{prox}_{\frac{1}{\gamma}f_{1}}(Bx_{k+1}+\frac{v_{k}}{\gamma}), \\ {v}_{k+1}=v_{k}+\gamma(Bx_{k+1}-d_{k+1}). \end{cases} $$

Checking the convergence condition of the theorem, if $\frac{\alpha}{\gamma}>\|B\|^{2}_{2}$, $\alpha>0$, and $\theta\in[0,1)$, we can easily get that the sequence ${x_{k}}$ generated from (54) converges to a solution of problem (33). We remark that when $\theta=1$, the scheme (54) reduces to the split Bregman method (51). When $\theta=0$, the scheme (54) is the preconditioned alternating method of multipliers as discussed in [2, 3]. Also, when *Q* is defined by (53) and $\rho\neq1$, the new primal-dual method can be expressed as

55 $$ \textstyle\begin{cases} \tilde{v}_{k}=(I+\gamma\partial f_{1}^{*})^{-1}(v_{k}+\gamma Bx_{k}), \\ \tilde{x}_{k}=(A^{T}A+\gamma\theta B^{T}B+\alpha(1-\theta)I)^{-1}((\gamma \theta B^{T}B+\alpha(1-\theta)I)x_{k} \\ \hphantom{\tilde{x}_{k}={}}{}-B^{T}(2\tilde{v}_{k}-v_{k})+A^{T}b), \\ x_{k+1}=x_{k}-\rho(x_{k}-\tilde{x}_{k}), \\ v_{k+1}=v_{k}-\rho(v_{k}-\tilde{v}_{k}). \end{cases} $$

The other positive definite matrix *Q* may be chosen as

56 $$ Q= \left ( \textstyle\begin{array}{@{}c@{\quad}c@{}} \frac{1}{\gamma}I&B\\ B^{T}& \frac{1}{\tau}I+\gamma B^{T}B \end{array}\displaystyle \right ). $$

By a simple manipulation we obtain

57 $$ \textstyle\begin{cases} x_{k+1}=(A^{T}A+\gamma B^{T}B+\frac{1}{\tau}I)^{-1}(\frac{1}{\tau}x_{k}+A^{T}b+ B^{T}(\gamma d_{k}-v_{k})), \\ d_{k+1}=\operatorname{prox}_{\frac{1}{\gamma}f_{1}}(Bx_{k+1}+\frac{v_{k}}{\gamma}), \\ {v}_{k+1}=v_{k}+\gamma(Bx_{k+1}-d_{k+1}). \end{cases} $$

According to [28], the eigenvalues of the matrix $B^{T}B$ all lie in the interval $[0,8)$. So, to guarantee the positive definite of *Q*, we should set $\gamma\tau >0$. In fact, the scheme (57) is the third case of Algorithm 2 in [17]. Finally, similarly to the previous subsection, we can also get a new relaxed splitting Bregman algorithm when $\rho\neq 1$ and *Q* is given by (56). Then, the new primal-dual algorithm can be reformulated as

58 $$ \textstyle\begin{cases} \tilde{v}_{k}=(I+\gamma\partial f_{1}^{*})^{-1}(v_{k}+\gamma Bx_{k}), \\ \tilde{x}_{k} =(A^{T}A+\gamma B^{T}B+\frac{1}{\tau}I)^{-1}((\frac{1}{\tau }I+\gamma B^{T}B) x_{k}-B^{T}(2\tilde{v}_{k}-v_{k})+A^{T}b), \\ x_{k+1}=x_{k}-\rho(x_{k}-\tilde{x}_{k}), \\ v_{k+1}=v_{k}-\rho(v_{k}-\tilde{v}_{k}). \end{cases} $$

## Conclusions

We proposed a primal-dual-based contraction framework in the prediction-correction fashion. The convergence and convergence rate of the proposed framework are also given. Some well-known algorithms, for example, the linearized primal-dual method, SIU, Bregman operator splitting method, and split Bregman method can be considered as particular cases of our algorithm framework. Some new primal-dual schemes such as (48), (52), (55), and (58) are induced. Finally, how to choose the adaptive parameter *ρ* is an interesting problem, which will be discussed in a forthcoming work.
